# Adsorption Properties and Wettability of Ethoxy- and Propoxy- Derivatives of 2-Ethylhexanol as Sterically Specific Surfactant Structures

**DOI:** 10.3390/molecules29030690

**Published:** 2024-02-02

**Authors:** Wiesław Hreczuch, Beata Konopczyńska, Marcin Stasiak, Adam Andrzejewski, Krystyna Prochaska

**Affiliations:** 1 MEXEO, Energetyków 9, 47-225 Kędzierzyn-Koźle, Poland; wieslaw.hreczuch@mexeo.pl; 2 Institute of Chemical Technology and Engineering, Poznan University of Technology, Berdychowo 4, 60-965 Poznań, Poland; beata.konopczynska@put.poznan.pl (B.K.); adam.andrzejewski@put.poznan.pl (A.A.); 3 Institute of Mathematics, Poznan University of Technology, Piotrowo 3, 60-965 Poznań, Poland; marcin.stasiak@put.poznan.pl

**Keywords:** 2-ethylhexanol, ethoxylation, propoxylation, surfactants, adsorption, micellization, diffusivity, wettability

## Abstract

2-ethylhexanol, an oxo alcohol competitively priced on the global market, has not been explored intensively as a raw material for surfactants, due to its weak hydrophobic character. However, its sequenced propoxylation and ethoxylation yield an innovative amphiphilic structure, which exhibits unique interfacial activity. The paper presents the differences in the fractional composition of innovative surfactants derived from 2-EH alcohol prepared using alkali and dimetalcyanide catalysts, as well as examples of excellent adsorption and interfacial properties of the latter. The adsorption behavior of the synthesized compounds was explored using equilibrium surface tension (the du Noüy ring method), dynamic surface tension (the maximum gas bubble pressure method) and static/dynamic contact angle (the sessile drop method). The results from the adsorption tests conducted at the air/aqueous surfactant solution interface underwent comprehensive qualitative and quantitative analyses. Moreover, based on the experimentally obtained dynamic surface tension isotherms and the developed algorithm, the diffusion coefficients for these preparations were estimated, and it was shown that the diffusivity of these surfactants is higher compared to the commercial formulations. The study’s outcomes in the testing of wettability indicate that new synthesized nonionic and anionic surfactants constitute an interesting group of amphiphiles with a wide application potential as effective wetting agents, especially in relation to the polymer surface. It should therefore be emphasized that the innovative surfactants described in this article, derived from 2-EH alcohol and prepared using dimetalcyanide catalysts, can successfully compete with conventional preparations such as ABS (Dodecylbenzenesulfonic Acid) or AES (Alcohol Ethoxysulphate) acid salts.

## 1. Introduction

**Figure 1:**
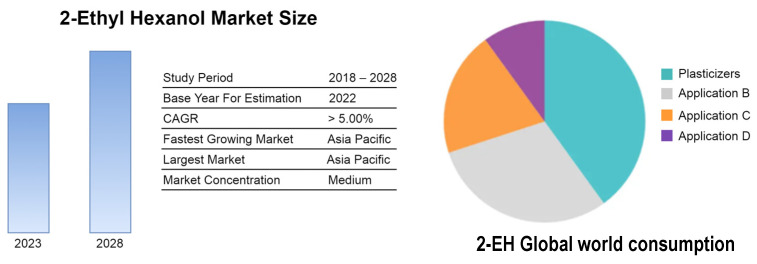
2-Ethylhexyl alcohol market size and global consumption (%) by application in: Plasticizers, B—EH Acrylate, C—EH Nitrate, D—Other applications (data from 2022) (Source: Mordor Intelligence; https://www.mordorintelligence.com/industry-reports/2-ethylhexanol (accessed on 14 January 2024)).

2-ethylhexanol (2-EH) ethoxylates have not been widely used as nonionic surfactants in part due to their inadequate amphiphilic properties. Their share in the production of ethoxylates is assessed to be less than 1%, suggesting values near zero. This observation aligns with global consumption data for 2-EH, where surfactants are not distinctly categorized (Figure 1).

As the result of radical reduction in application of the 2-EH-derived phthalate plasticizers, with significant spare capacities of 2-ethylhexanol, there is a notable availability for new applications at competitive prices. 2-Ethylhexanol, being a mass-produced synthetic organic raw material in Europe, including Poland, holds particular interest for the detergent industry due to its undeniable economic competitiveness, provided its utility is established. 

Recent studies have examined numerous critical aspects concerning the synthesis and interfacial activity of the discussed oxyethylene derivatives of 2-ethylhexanol, showing positive results [1,2,3,4,5].

Our preliminary research on and applications for the sterically specific, polydisperse surfactants in question demonstrated particular potential in the use of their superior wetting properties and in applications for modern oil-free lubricating products or in other preparations of detergent super-concentrates with spontaneous water solubility and devoid of troublesome gelation phases. The anionic sulfosuccinate derivatives described in this article can also successfully compete with conventional ABS or AES acid salts.

The premises encouraging the development of practical applications of the discussed surfactants are their extremely high biodegradability, confirmed by standard tests, and potentially lower ecotoxicity, resulting from the relatively lower content of low-oxyethylene homologs, compared to their conventional alkyl ether counterparts, which was also demonstrated in this work.

The aim of this article is to present new results regarding the evaluation and comparison of the surface activity and wettability of selected non-ionic oligo-oxyethylates of 2-ethylhexanol and its monopropoxylene derivatives as well as their sulfosuccinate esters as anionic surfactants against the background of these properties concerning well-known reference surfactants.

## 2. Results and Discussion

### 2.1. Sterically Specific Surfactants

**Figure 2:**
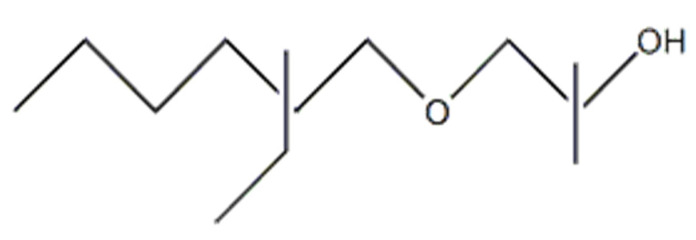
Chemical structure of the exemplified sterically specific molecule of l-(2-ethylhexyloxy)propane-2-ol.

The idea of the new approach involves the addition of an oxypropylene part to 2-ethylhexanol. This modification enhances its lipophilicity and imparts a sterically specific type of double-branched alkyl-ether structure (Figure 2). The subsequent polyoxyethylation of this modified structure results in a higher conversion rate, leading to surfactants with unique surface activity and functional properties.

**Figure 3:**
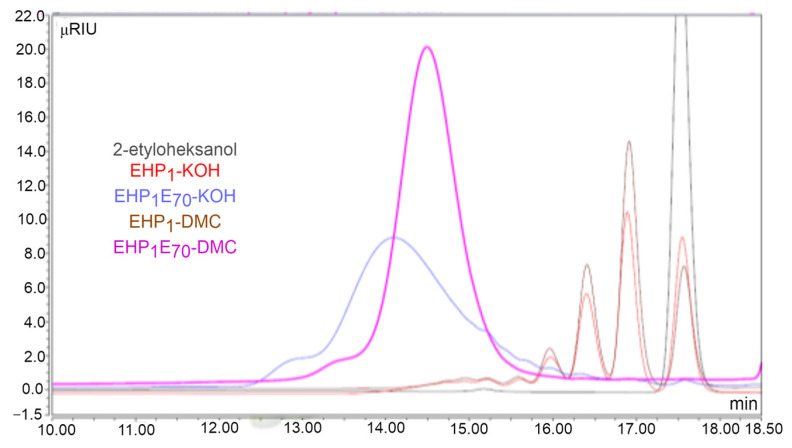
GPC chromatogram of individual EH, mono-propoxylated EH (EHP_1_), and *n* = 9 oxyethylate of mono-propoxylated 2-EH (EHP_1_E_9_), as obtained with DMC and KOH catalysts.

In this study, the oxyalkylation process was conducted in the presence of two types of catalysts. The resultant polydisperse products exhibited distinct characteristics, including a narrow (DMC-promoted) and wide (with KOH) distribution of homologues, respectively. The polydispersity of the resulting reaction products makes quantitative determination of the composition virtually impossible due to the complex, multifunctional nature of these oxyalkylene derivatives and their high molecular weights. Gel permeation chromatography was employed as a useful semi-quantitative method for analyzing these products. The studied EHP_a_E_n_. products are exemplified in Figure 3, which illustrates a comparative summary of the chromatograms obtained during the synthesis of EHP_1_E_9_ derivatives using both DMC and KOH catalysts.

Previous studies, as referenced above, have established that dimetalcyanide catalysts, in addition to their extraordinary kinetic activity, generate a narrow distribution of homologues of polydispersive oxyalkylation products, as compared to their equivalents produced with the conventional KOH catalyst. This phenomenon is evident in both oxyethylation and oxypropylation processes.

As illustrated in Figure 3, the use of a DMC catalyst significantly alters the homologue distribution even at the mono-propoxylation stage, resulting in enhanced conversion of 2-EH and a higher concentration of P1 to P3 homologues in the final product. Further polyethoxylation brings a more spectacular effect of narrowing the distribution of DMC-based products.

The observed fundamental variation in the distribution of homologues, contingent upon the catalyst used, will translate in favor of a narrower range of molecular weights in all equivalents of polyoxyalkylates prepared with DMC catalysts compared to KOH. As a result, different surfactant performances can be expected, depending on the polyaddition degree as well as the applied catalyst.

**Figure 4:**
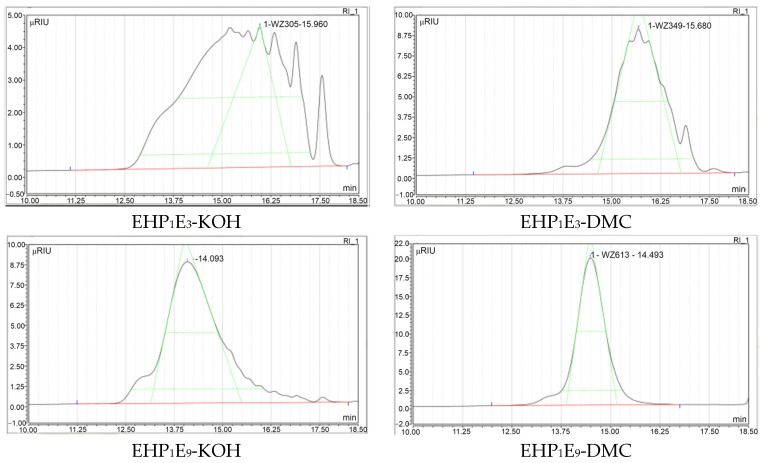
GPC chromatograms of exemplary EHP_1_E_3_ and EHP_1_E_9_ EH oxyalkylates, depending on the applied catalyst, DMC or KOH (black lines stand for chromatogram peak, red lines are peak baselines and green lines are tangent lines, to measure peaks using triangulation method).

Figure 4 demonstrates the variation in product composition depending on the average degree of polyaddition as exemplified by samples with an ethoxylation degree of *n* = 3 and *n* = 9 and as obtained in the presence of DMC catalysts and KOH, respectively (Figure 4).

Figure 4 reveals that the width of the peaks quantitatively reflecting the range of molecular weights of the generated homologues, depending on the catalyst used, is considerably narrower for the reaction involving the DMC catalyst as compared to KOH. The discussed range of average molecular weights of counterparts with the same average polyaddition degree, depending on the catalyst used, is quantified in Table 1.

The scatter of generated molecular masses of homologues is an important quality parameter of polydisperse reaction products, also known as their polydispersity. It is often measured by the MWD = M_W_/M_N_ value, where M_W_ = ∑N_i_M_i_^2^/∑N_i_M_i_ and denotes the weight average molar mass and M_N_ = ∑N_i_M_i_/∑N_i_ is the number average of molar mass; these are well-known characteristics in the polymerization area. A narrow dispersion, defined by a MWD value close to 1, is usually desirable, indicating greater homogeneity of the product’s macromolecular masses. The lower MWD values of the compared polyoxyalkylation products obtained with the DMC catalyst reflect the narrower molar mass ranges seen in the chromatograms in Figure 4 and in Table 2 for these products, as compared to those promoted by KOH. Furthermore, the polydispersity of the tested products decreases when increasing the average molecular weights.

The distinctly different molecular weight range of the homologues, depending on the product’s average polyaddition degree and the applied catalyst, is further illustrated at half of the peak height (Table 1). This parameter decreases with an increasing degree of polyaddition and is more pronounced in products synthesized with the DMC catalyst compared to those with KOH.

Owing to the higher conversion of the starter alcohol and the narrower polydispersity of the homologues, ethylhexanol oxyalkylates and their sulfosuccinate derivatives obtained with the DMC catalyst were further investigated for surface activity and wettability.

### 2.2. Adsorption Properties

#### 2.2.1. Equilibrium Surface Tension Isotherms

**Figure 5:**
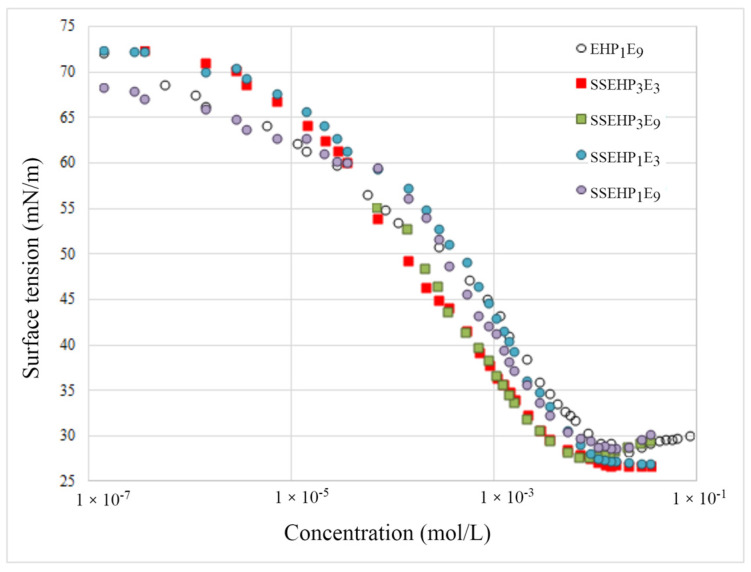
Surface tension isotherms for synthesized nonionic and anionic surfactants derived from 2-EH alcohol.

Figure 5 presents the surface tension (γ) versus log (concentration) profiles at 25 ± 0.1 °C for all synthesized surfactants.

The experimentally obtained surface tension isotherms for all synthesized surfactants derived from 2-EH have a typical course for amphiphilic compounds. It is evident that, with an increase in the concentration of surfactant in the tested solution, a linear decrease in the measured surface tension (γ) is visible, up to a specific concentration, above which the surface tension remains practically unchanged. A slight increase in surface tension above cmc, observed especially in the case of isotherms obtained for 2-EH derivatives with longer oxyethylene chains (EHP_1_E_9_, SSEHP_1_E_9_), is the result of the greater polydispersity of these surfactants. The cmc concentration corresponds to the minimum concentration of the compound above which it self-aggregates. The effect of aggregation in aqueous solutions is the formation of micelles. This concentration, called the critical micelle concentration (CMC), is a measure of micelle stability [6,7,8]. The CMC values estimated for the tested 2-EH derivatives based on the analysis of the course of experimentally determined surface tension isotherms are presented in Table 2.

The qualitative analysis of the course of the experimentally determined dependence γ = f(lg(c), presented in Figure 5, shows that all isotherms have a similar course, which means that the tested derivatives are characterized by a similar ability to adsorb at the air/water interface and reveal a similar ability for micellization after saturation of the interface. It may seem quite surprising that there is essentially no significant difference in the nature of the surface tension isotherm determined for the non-ionic EHP_1_E_9_ derivative and the SS EHP_1_E_9_ anionic surfactant [9,10]. It should be noted, however, that the differences of the adsorption properties of individual derivatives becomes visible only after a detailed quantitative analysis of the determined surface tension isotherms is performed.

In order to quantify the adsorption properties of the synthesized derivatives, the pC_20_ surface tension lowering efficiency was estimated (Table 3), defined as the negative logarithm of the surfactant concentration at which the surface tension of a given solvent was reduced by 20 mN/m. The higher the pC_20_ value, the stronger the surface properties of a given surfactant [11,12].

Another parameter of the quantitative evaluation of the adsorption properties of surfactants is the surface tension lowering efficiency Π_CMC_, the measure of which is the difference between the value of the initial surface tension γ_0_ and the value achieved at the CMC. The higher the Π_CMC_ value, the higher the surface tension lowering efficiency of a given surfactant [10,13].

**Figure 6:**
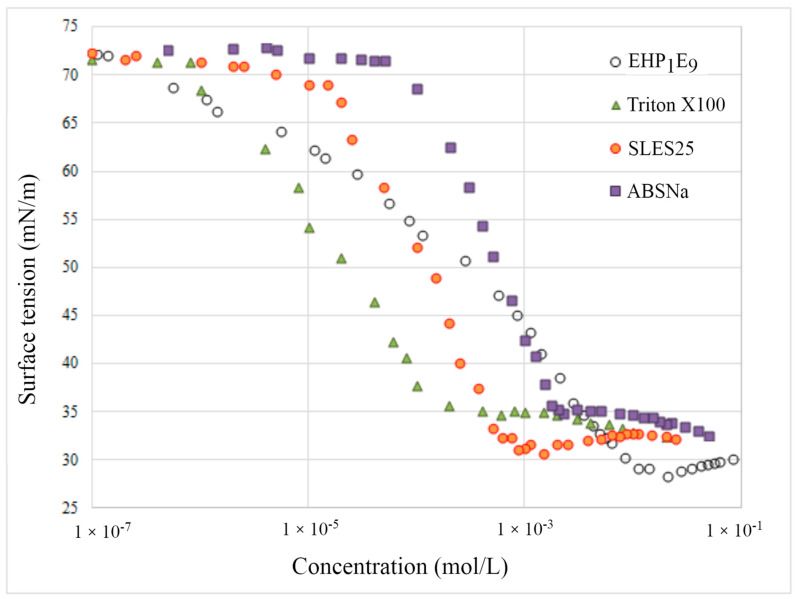
Surface tension isotherms for the commercial surfactants and EHP_1_E_9_ derivative.

In order to compare the adsorption properties of the synthesized compounds with commercial, commonly used surfactants, Figure 6 compares surface tension isotherms for the additional three commercial preparations: SDS, SLES25, and Triton X100. The analysis of the isotherm course (as well as the data tabulated in Table 1) shows that the synthesized EHP1E9 derivative exhibits a higher CMC value compared to the considered surfactants. However, it is noteworthy that at a concentration of CMC, the non-ionic surfactant EHP1E9 lowers the surface tension of water more effectively than the other three commercial formulations. Moreover, a comparison of the magnitude of the adsorption parameters (Table 3) estimated for commercial ABSNa and SLES25 surfactants and the synthesized nonionic and anionic surfactants derived from 2-EH alcohol shows that the commercial preparations reveal lower tendency to adsorb at the air/aqueous solution interface [14,15,16]. The ΔG_ads_ values of the synthesized derivatives are much smaller. For instance, the value of free energy of adsorption estimated for the anionically active commercial compound ABSNa is over 50% higher compared to the ionic derivative SSEHP1E9. Additionally, it is observed that commercial formulations form a much more densely populated adsorption monolayer at the saturated interface. Values of Γ^∞^ estimated for commercial preparations are two to three times higher than in the case of synthesized derivatives, both ionic and non-ionic.

**Figure 7:**
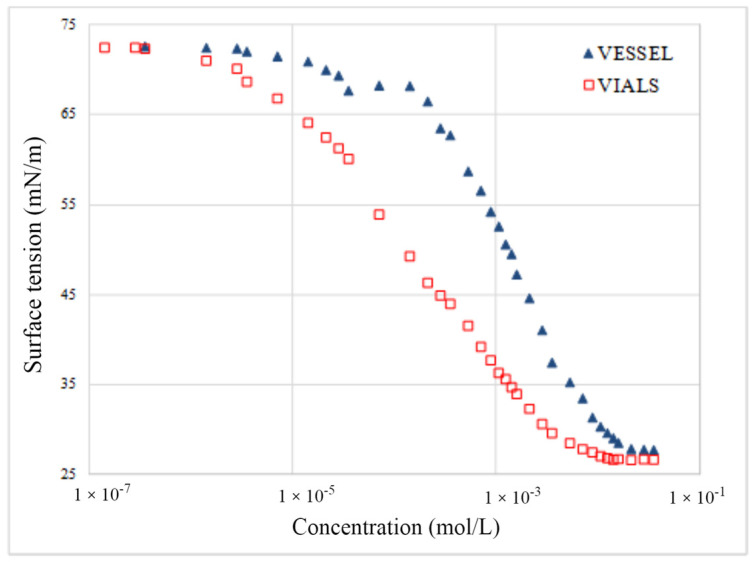
Effect of solution dilution methodology on the run of surface tension isotherms for SSEHP_3_E_3_, where: “vessel”—diluting the solution to a given concentration directly in the measuring cuvette; “vials”—solutions prepared by successive dilutions in separate flasks before measurement.

An important distinction to be made between the commercial surfactants and the synthesized 2-EH alcohol derivatives lies in the sterically specific character of their double-branched hydrophobic structure. As Figure 7 demonstrates, the course of the surface tension isotherm significantly varies depending on the method of diluting the solution. There are differences of up to 20 mN/m in the measured surface tension values, contingent on the solution’s concentration. The surface tension of the solution at a concentration of 10^−3^ mol/L, which was diluted directly in the measuring cuvette, is 45 mN/m. However, when the solution before the measurement is prepared by successive dilutions in separate flasks, the value of γ is much greater and equal to about 65 mN/m. In the case of all other synthesized derivatives, a strong influence of the dilution method of the stock solution on the measured value of the surface tension was also observed.

**Figure 8:**
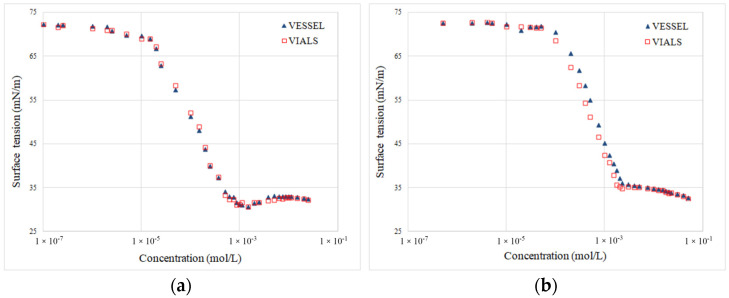
Effects of solution dilution methodology on the run of surface isotherms for typical linear amphiphiles: (**a**) SLES25 and (**b**) ABSNa.

The observed phenomena can be explained by superior absorption of the sterically specific amphiphilic structure at the glass/water interphase, which significantly reduced the concentration (availability) of these surfactants in the solution [17,18,19]. The above-mentioned effect is practically not observed with a typical linear amphiphile, such as SLES25 or ABSNa (Figure 8).

#### 2.2.2. Study of Dynamics of Adsorption

**Figure 9:**
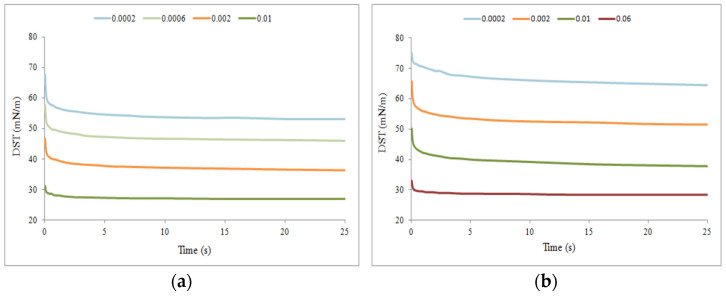
Dynamic surface tension isotherms for (**a**) EHP_1_E_9_ and (**b**) SSEHP_1_E_9_ derivative (the concentration is expressed in mol/L).

Experimentally obtained dynamic surface tension isotherms for EHP_1_E_9_ and SSEHP_1_E_9_ derivatives are shown in Figure 9. Selected curves highlight the adsorption dynamics at different phases of the process. These time-dependent curves generally resemble exponential functions, with the base in the range from 0 to 1. Thus, the rapid asymptotic decrease to the plateau was observed for each curve. The time needed to reach the equilibrium value of the surface tension in a given solution practically does not depend on the surfactant concentration. Both at low concentration (i.e., below CMC) and in the micellar solutions (i.e., above CMC), in a very short time, not more than 5 s, the systems achieve a constant surface tension value [20,21]. Thus, for every concerned surfactant and its concentration, both the diffusion process to the air/water interphase and adsorption process at the air/water interphase were almost immediate.

Despite these similarities, differences are also visible between the DST for the non-ionic surfactant (Figure 9a) and its ionic derivative (Figure 9b). While the general trends are similar, the curves for individual concentration values differ due to the ionic nature of the SSEHP1E9 derivative. DST plateau values for the ionic surfactant are higher compared to the non-ionic surfactant due to electrostatic interactions, i.e., the presence of charges, which can affect the packing of surfactant molecules at the interface and interactions with the surrounding medium (water).

The experimentally determined dynamic surface tension isotherms allowed determination of the thermodynamic stability of micelles formed in micellar solutions by the anionic and non-ionic surfactants derived from the 2-EH alcohol as well as estimation of the values of diffusion coefficients for the compounds considered.

##### Micelle Stability

Based on the research on the dynamics of adsorption in micellar solutions, in accordance with the relationship (17), the values of the stability constants of the micelles were estimated and are summarized in Table 3. In addition, Table 2 shows the values of **τ**_2_, i.e., the slow relaxation time process, related to the change in the number of aggregations of spherical micelles. The obtained results indicate that in the case of almost all synthesized derivatives, the k_2_ values are less than 1 s^−1^ and vary within a very small range of 0.5–0.91 s^−1^, which means that for the synthesized surfactants, the time of the slow relaxation process of the micelle kinetics ranges from about 2 to about 1 s. Only in the case of the SSEHP_3_E_3_ derivative is the estimated time of the slow relaxation process of the micelle kinetics greater, in the order of 1.6 s, similar to the commercial compound Triton X100.

On the other hand, it was observed that in the case of the synthesized anionic derivatives, the higher the CMC, the lower the relaxation time, i.e., the higher the k_2_ value. This finding is consistent with the literature [22,23,24,25].

#### 2.2.3. Diffusion Coefficients

The process of adsorption of surfactants at the gas/liquid interface involves two mechanisms—the diffusion of molecules to the interphase and their adsorption on the surface. In systems with surfactants, a monolayer is formed at the interface, in which the electrostatic interactions are usually ignored [26,27]. When the process of diffusion of molecules to the interphase is slower than the adsorption of these molecules, the diffusion seems to determine the speed of the entire process. In other words, the adsorption process is diffusion controlled. The process model described above is given by the one-dimensional diffusion equation as specified in Equation (1) [28,29,30,31].

(1) $$\frac{\partial c}{\partial t}=D\frac{\partial^{2}c}{\partial x^{2}}$$

(2) $$D\frac{\partial c}{\partial x}{|}_{x=0}=\frac{\partial \Gamma }{\partial t}$$

(3) $$c\left(x,t\right)=c_{b}$$

with boundary conditions (8) and (9):

(4) $${c\left(x,t\right)|}_{t=0}=c_{b}$$

for *t* > 0, where *c_b_* is a bulk concentration, and the initial condition is (4).

In 1946, Ward and Tordai [32] published the solution to the diffusion model, which describes the dynamic of surfactant adsorption over long time periods, as expressed in Equation (5) [33].

(5) $$\Gamma \left(t\right)=\sqrt{\frac{D}{\pi }}\left\{\left.2c_{b}\sqrt{t}-\int_{0}^{t}\frac{c(\Gamma \left(\tau \right))}{\sqrt{t-\tau }}d\tau \right\}\right.$$

where *D* (m^2^/s) is a diffusion coefficient, *c(*Γ*(t))* is the relation between concentration and surface excess, and *t* is time.

Equation (5) is a Volterra integral equation of the second kind. The linearity or non-linearity of this equation relies on the adopted adsorption isotherm model. Sutherland solved analytically the linear case by applying Henry’s isotherm [34,35]. In this paper, the Langmuir isotherm (6) is inserted into the Ward–Tordai equation [10]. Such a non-linear case does not have an analytical solution.

(6) $$\Gamma =\Gamma_{\infty }\frac{K_{L}c}{1+K_{L}c}$$

Based on data from equilibrium and dynamic adsorption studies, the necessary parameters of the tested surfactants were determined in order to solve the given problem numerically using the following steps.

The Gibbs isotherm, given by Equation (7), allows for the calculation of the equilibrium value of surface excess Γ*_eq_* (mol/m^2^) of the proposed system.

(7) $$\Gamma_{eq}=-\frac{1}{RT}\frac{d\gamma }{dlnc}$$

where *R* (J/(mol·K)) is a gas constant, *T* (K) is the temperature of the system, and *γ* (mN/m) is the surface tension of the system [36].

**Figure 10:**
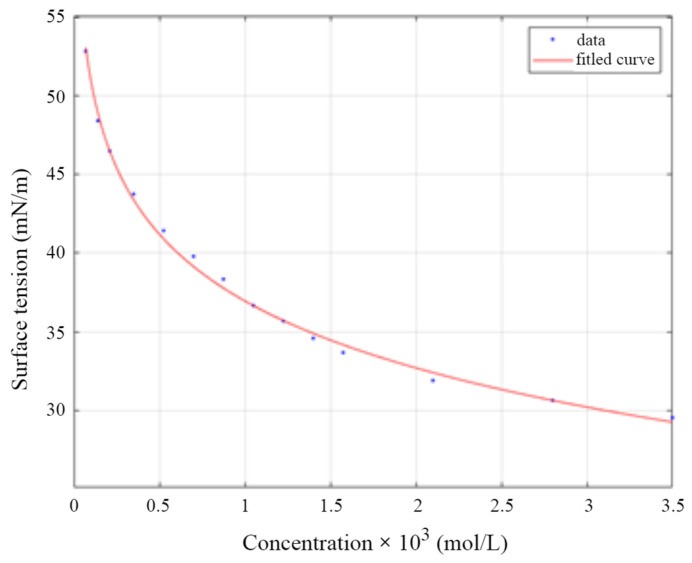
Szyszkowski isotherm fitted to the experimental data of the surface tension vs. bulk concentration for SSEHP_3_E_9_ derivative.

The Szyszkowski isotherm, as shown in Equation (8), was applied to the experimental data of the surface tension as a function of the bulk concentration of the compound (Figure 10) [8].

(8) $$\gamma =\gamma_{0}\left(1-B_{sz}ln\;ln\;\left(\frac{c}{A_{sz}}+1\right)\right)$$

where *γ*_0_ is the water surface tension, *γ* is the surface tension associated with the surfactant concentration *c*, *A_sz_* and *B_sz_* are Szyszkowski constants, which have physical meanings. *A_sz_* describes a measure of the tendency toward interfacial adsorption, and *B_sz_* characterizes the orientation of the adsorbed molecule.

The values of Γ*_eq_* and Γ*_∞_* of the considered system were determined using Equations (7) and (8). A novel approach proposed in this research for the approximation of adsorption dynamics is the use of cubic splines as an alternative to the power function commonly used in the literature. This method allows for a more accurate approximation of discrete experimental data.

The relation between surface excess and surface tension is given by Equation (3), which was utilized to calculate values of the surface excess Γ as a function of time [35,36,37].

(9) $$\Gamma \left(t\right)=\Gamma_{\infty }\left(1-exp\;exp\;\left(\frac{\gamma \left(t\right)-\gamma_{0}}{RT\Gamma_{\infty }}\right)\right)$$

In the next step, the diffusion coefficient of the tested surfactant is calculated based on the values of surface excess change over time. The values of the diffusion coefficient were investigated in the range from 10^−16^ to 10^−7^ (m^2^/s). In order to solve the non-linear Ward–Tordai integral equation associated with the Langmuir isotherm, the Nystrom method was applied [38,39,40]. Equation (5) was discretized, and its final form is given by Equation (10), with the initial step Γ(0) = 0, which means a lack of any surfactant molecule adsorbed on the surface at the beginning of the diffusion process.

(10) $$\Gamma \left(t_{i}\right)=2c_{b}\sqrt{\frac{Dt_{i}}{\pi }}-\frac{1}{K_{L}}\sqrt{\frac{D}{\pi }}\sum_{j=1}^{i}A_{j}\frac{\Gamma \left(\tau_{j}\right)}{\sqrt{t_{i}-\tau_{j}}\left(\Gamma_{\infty }-\Gamma \left(\tau_{j}\right)\right)}$$

The golden-section search was used in the optimization part of the inverse problem algorithm. The objective function defined by Equation (11) is the maximum norm of the subtraction of the experimental and numerical data vectors, Γ*_num_* and Γ*_exp_*, respectively.

(11) $$\Psi (D)=\|\Gamma_{num}-\Gamma_{exp}\|$$

**Figure 11:**
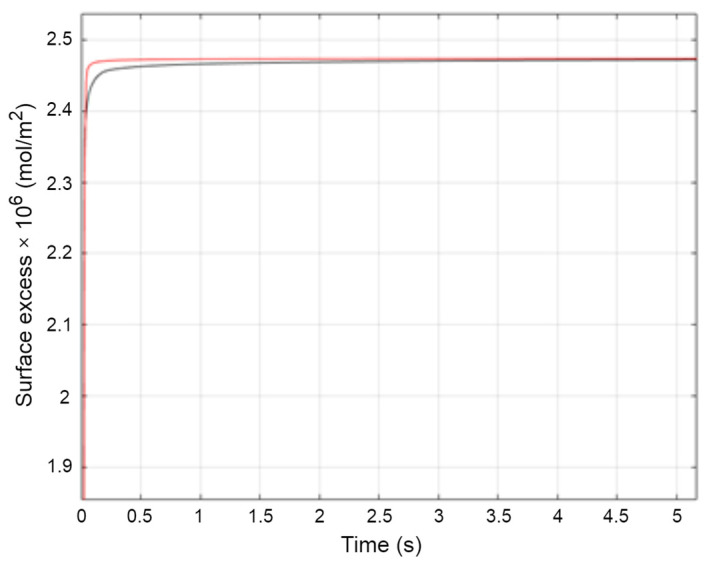
Experimental data (**––**) of surface excess and numerical approximation (**––**) for SSEHP_3_E_9_ concentration 8.75 × 10^−4^ mol/L.

The experimental data were compared with the obtained results of the numerical calculations; the error was minimized successfully in a numerical inverse problem algorithm for almost all considered samples (Figure 11).

**Figure 12:**
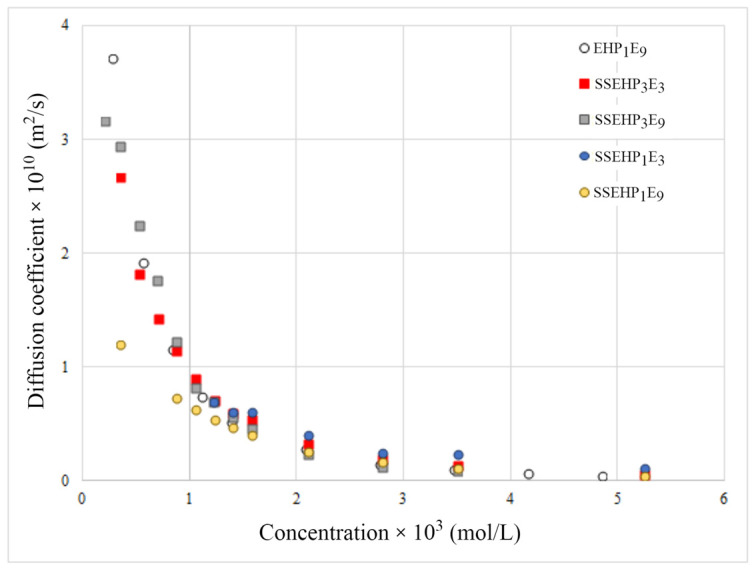
Dependence of the diffusion coefficient vs. concentration for nonionic and anionic surfactants derived from 2-EH alcohol.

Figure 12 illustrates the dependence of the diffusion coefficient as a function of concentration for the considered nonionic and anionic surfactants derived from the 2-EH alcohol. Each data point in Figure 12 represents a solution of the developed numerical inverse problem algorithm for a specific concentration of the investigated surfactant.

**Figure 13:**
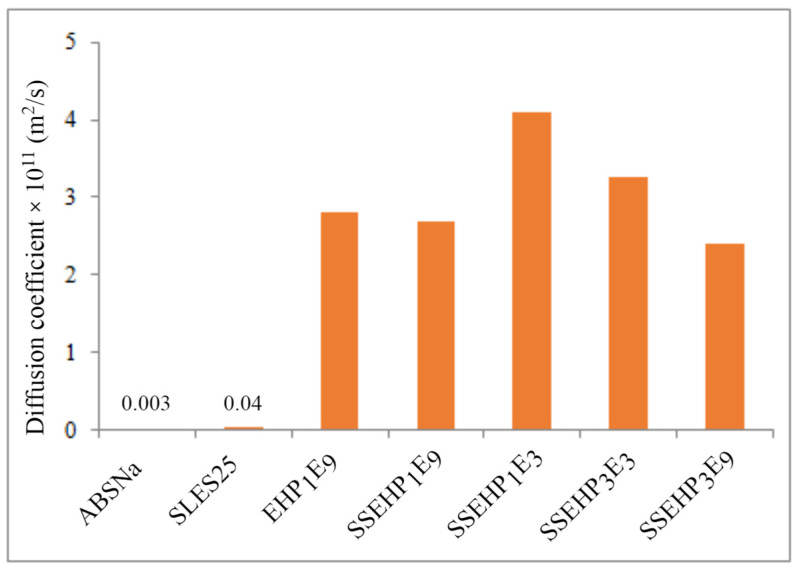
Comparison of diffusion coefficient for surfactants derived from 2-EH alcohol and commercial preparations.

The result of the presented numerical studies is the diffusion coefficient dependence on the bulk concentration of the surfactant, which is particularly pronounced in the case of the nonionic derivative. As one can see, the diffusivity of new surfactants is approximately two to three orders of magnitude higher compared to the commercial preparations (Figure 13). On the other hand, it is worth adding that the presented algorithm can be successfully used to determine the diffusion coefficient of surfactants based on the experimental data of the dynamic adsorption process.

#### 2.2.4. Wettability Properties

**Figure 14:**
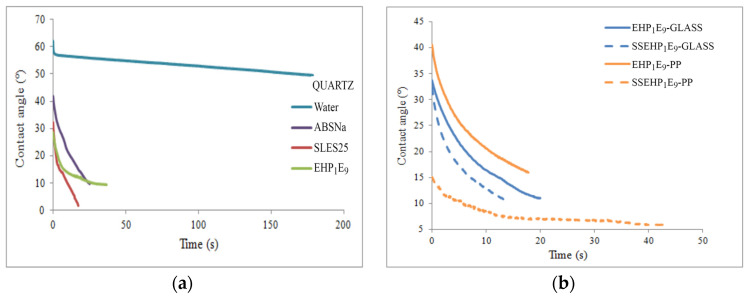
The comparison of dynamic contact angle curves for (**a**) commercial surfactants and nonionic derivative EHP_1_E_9_ and (**b**) anionic and nonionic synthesized derivatives of 2-ethylohexanol.

Figure 14 displays the contact angle dynamic curves for exemplary commercially available surfactants and nonionic and anionic synthesized derivatives of 2-EH.

**Figure 15:**
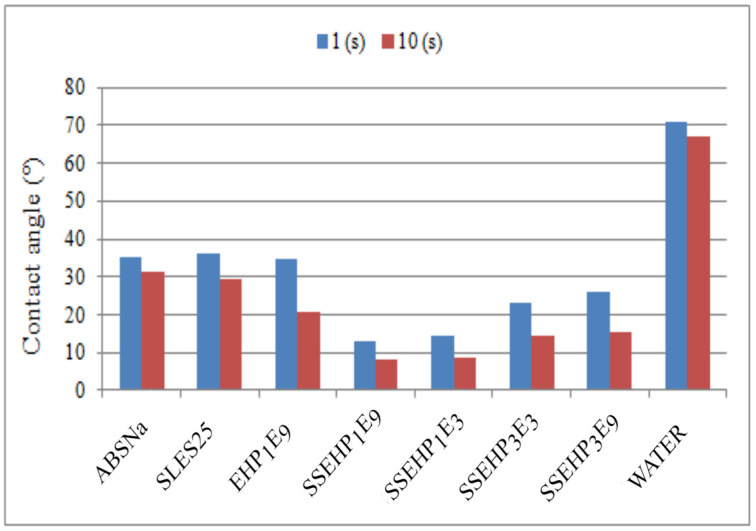
The comparison of contact angle on polypropylene surface at 2 and 10 s for commercial surfactants and nonionic and anionic synthesized derivatives of 2-ethylohexanol.

In this study, it is noteworthy that all surfactants considered have better wetting properties than pure water. However, the material of substrate also significantly influenced the wetting properties of the tested surfactants. As evidenced in Figure 14, the wettability of the polypropylene surface was better compared to the glass material, particularly in the case of the anionic derivative. Notably, for the synthesized anionic compounds, the wettability of the tested polymeric material was significantly better compared to commonly used surfactants (Figure 15). Consequently, it can be inferred that the synthesized derivatives of 2-ethylohexanol belong to the group of highly effective wetting agents.

## 3. Materials and Experimental Methods

### 3.1. Materials

The focus of this research was the newly synthesized, by the authors, 2-EH alcohol derived nonionic and anionic surfactants, as specified in Table 4, and the following commercial amphiphilic compounds: Triton X100, 4-(1,1,3,3-Tetramethylbutyl)phe- nyl-polyethylene glycol, *t*-Oct-C_6_H_4_-(OCH_2_CH_2_)_n_OH, *n* = 9 or 10; 100% purity (declared by the manufacturer, Merck, Poznań, Poland), SLES25 (α-Sulfo-ω-(dodecyloxy)-poly(oxyethane-1,2-diyl), sodium salt, CH_3_(CH_2_)_11_(OCH_2_CH_2_)*_n_*OSO_3_Na), active substance content 25.5 ± 1%, Brenntag, Kędzierzyn-Koźle, Poland; ABSNa (sodium dodecylbenzene-sulfonate, CH_3_(CH_2_)_11_ C_6_H_4_SO_3_Na, active substance content 50 ± 2% (PCC Exol, Brzeg Dolny, Poland) and SDS (sodium lauryl sulfate, CH_3_(CH_2_)_11_OSO_3_Na, purity 99.0%, Sigma-Aldrich, Poznań, Poland).

The following raw materials were used for the syntheses:

- 2-ethylhexanol of purity 99.4% m/m, manufacturer GA ZAK Kędzierzyn-Koźle; before alkoxylation, the alcohol was dehydrated under nitrogen purge to a water content of 0.03% m/m.
- DMC catalyst, manufacturer MEXEO company, Kędzierzyn-Koźle, Poland.
- Maleic anhydride of purity 99.8% m/m, GA ZAK Kędzierzyn-Koźle, Poland.
- Sodium sulfite anhydrous with a purity of 94% m/m, from CHEMPUR, Piekary Śląskie, Poland.

### 3.2. Method of Synthesis

The concept of the proposed EHP_a_E_n_ surfactant series relates to problems that create conditions for meeting complex environmental, economic, and technological requirements for advanced surface active agents. The general availability of 2-EH, oxirane, and methyloxirane in the global market of raw materials determines their competitive prices. In addition, they are easily biodegradable, non-toxic to the environment, and not susceptible to hydrolysis. Additionally, it is possible to use the tested mono-propoxy-poly- oxypolyoxyethylene glycol 2-ethylhexanol ether for making sulfated derivatives or sulfosuccinates as anionic surface active agents, equivalents of popular alkyl sulfates or alkylethersulfates and alkylbenzene sulfonates. The summary reactions of the discussed synthesis of non-ionic and anionic surfactants are illustrated by the following equations and methods.

#### 3.2.1. Description of the Synthesis of the Studied Surfactants

Using 2-ethylhexanol as a starter material, oxyalkylation was performed with a new generation catalyst, dimetalcyanide (DMC), a non-stehiometric zinc hexocyanocobaltate salt produced at MEXEO, Kędzierzyn-Koźle [41], Poland, and in the presence of classical alkaline catalyst, KOH, respectively.

The obtained EHPaE_n_ block copolymers were converted into maleic acid monoesters. The obtained monoesters were subjected to sulfidation with sodium sulfite aqueous solution, synthesizing the samples of sodium sulfosuccinate salts at a concentration of 35%.

The process used to obtain oxyalkylates can be described by the following equations:

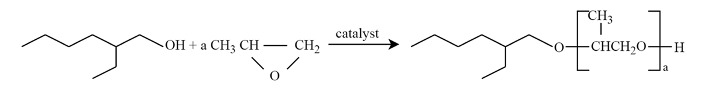
(12)
where: a = 1 or 3, catalyst = DMC


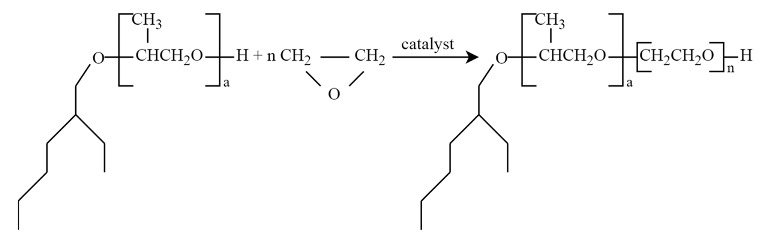
(13)
where *n* = 3 or 9, and the catalyst = KOH or DMC.

The syntheses were carried out with 2-ethylhexanol in the presence of KOH or DMC as catalysts.

Sulfosuccinic acid monoesters were obtained as follows:

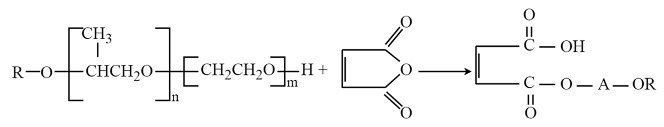
(14)
where R = 2-ethylhexyl, C_12_H_25_-, C_14_H_29_-; A is the alcoxy part.

The applied process to obtain the sodium salts of sulfosuccinates can be described by the following equation:

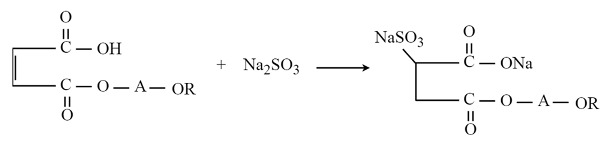
(15)
where R = 2-ethylhexyl, and A is the alcoxy part.

#### 3.2.2. Chromatographic Analysis

Analyses of the composition of the tested surfactants by the chromatographic technique were performed using the UltiMate 3000 ThermoScientific LPG 3400 SD UHPLC Chromatograph (Warszawa, Poland) equipped with a gradient pump, a Rheodine hand-held injector with a −20 μL loop, and a Refractomax 521 RI detector.

The following two gel chromatographic columns, connected in series, were used:

▪ Shodex GPC KF-802.5, 8 × 300 mm column (number of theoretical shelves ≥ 18,000; filling grain diameter 6μm; molecular weight range 300–8000 Da);
▪ Shodex GPC KF-801, 8 × 300 mm column (number of theoretical shelves ≥ 18,000; filling grain diameter 6μm; molecular weight range 100–700 Da).

Additionally:
Pre-column KF-G4A, Chromeleon 7.2. + GPC/SEC software, and the mobile phase Tetrahydrofuran, BAKER ANALYZED HPLC, J.T. Baker, were applied;
The sample solvent was THF, as above.
Calibration procedure: The molecular weight calibration was performed using the internal standard method based on the identified peaks of individual homologs of 2-ethylhexanol ethoxylates, whose GPC signals showed high quality in terms of resolution and thus detection and quantitative measurement of the surface area.

The molecular weight values (mass average) were calculated based on Equation (16):

log(M_w_) = −0.0083⋅(RT)^2^ + 0.0495⋅RT + 3.829 (16)

where M_w_—average molecular weight, Da; RT—retention time, min.

### 3.3. Experimental Methods and Calculations of Physicochemical Parameters

#### 3.3.1. Surface Tension

Tensiometry was used to evaluate the surface activity characteristics of the synthesized surfactants derived from 2-EH alcohol as well as commercial compounds.

Equilibrium surface tension at the air/water system for all surfactants considered was measured by a du Noüy ring method using a KSV Sigma 701 tensiometer (KSV, Helsinki, Finland) having an accuracy of ±0.01 mNm^−1^. Tensiometry was used to evaluate the surface activity characteristics of the synthesized surfactants derived from 2-EH alcohol as well as commercial compounds.

In research conducted using the fully automatic method, the pull-version was used. All measurements were carried out at 25 ± 0.1 °C with a water circulating thermostat. The temperature of all surfactant solutions was maintained at 25 ± 0.1 °C by a thermostatic water bath. Concentrated stock solutions of surfactants were freshly prepared in doubly distilled water and then diluted in the same medium to the appropriate concentration. For every concentration, the surface tension was measured three times and with an average deviation of less than 0.2 mNm^−1^.

Critical micelle concentration (CMC) of the synthesized amphiphiles were estimated from the surface tension vs. log(concentration) plots. Furthermore, the experimentally obtained points of surface tension were fit using the Szyszkowski isotherm equation, Equation (14), according to the method described in [42], which allowed estimation of the adsorption parameters, including the surface excess at the saturated interface (℘^∞^) and the Gibbs energy of adsorption (ΔG_ads_). All calculations were performed using IsoFit software [43].

Dynamic surface tension (DST) measurements were carried out by the maximum gas bubble pressure method using the SITA t60 apparatus (SITA Messtechnik GmbH, Dresden, Germany). The device allows for obtaining results as a relationship between the bubble lifetime and surface pressure. The changes in the surface tension were recorded in the 30 ÷ 60,000 ms time interval. DST measurements were carried out in a 35 mm diameter cuvette, in which the capillary was immersed in each tested surfactant solution to the same depth, equal to 1 cm.

On the basis of the DST results, graphs were drawn showing the dependence of the dynamic surface tension vs. the lifetime of the gas bubble. Then, on the basis of the time curves, the micelle stability constants were estimated according to the relationship (17) described in [44].

(17) $$\frac{{\left[\frac{d\gamma_{d}}{{dt}^{-\frac{1}{2}}}\right]}_{c\leq CMC}}{{\left[\frac{d\gamma_{d}}{{dt}^{-1}}\right]}_{c>CMC}}=\alpha {\left(\frac{k_{2}\pi }{4}\right)}^{\frac{1}{2}}$$

where *k*_2_—micelle stability constant, which is inversely proportional to |_2_, i.e., the slow time of the relaxation process of micelle kinetics, *k*_2_ = |_2_^−1^; *γ_d_*—dynamic surface tension; *t*—time of gas (air) bubble life; *α*—value relative concentration of monomers in solutions with concentrations higher than the CMC value, in relation to c = CMC.

The derivative *dγ*/*dt*^−1/2^ was determined from the *γ* dependence on *t*^−^^1/2^ for *t*→∞ at c = CMC, and the derivative *dγ*/*dt*^−1^ was determined from *γ* dependence on *t*^−^^1^ for *t*→∞ at concentrations above CMC.

#### 3.3.2. Measurement of Wettability

Contact angle measurements were performed using the sessile drop method. Wettability tests were carried out for aqueous solutions of individual surfactants of various concentrations, employing a Theta optical tensiometer (Attension, KSV Finland). All measurements were performed at a temperature of 25 ± 0.1 °C. The droplets were automatically pushed out of the capillary and deposited on the surface of the tested material. Three different model surfaces were used in the research: glass, quartz, and polypropylene. In all measurements, the volume of the drops was the same: 3 µL. The shape of the drops after being applied to a solid substrate was observed using a video camera. The strategy used was to match the experimental dip meridian to the theoretical dip profile according to the Young–Laplace equation. The measurements were repeated three times for each sample, and the average contact angle values were reported.

## 4. Conclusions

The presented results allow for comparison of the surface activity and wettability of new synthesized non-ionic oligo-oxyethylates of 2-ethylhexanol as well as their sulfosuccinate esters as anionic surfactants against the background of these properties concerning well-known reference surfactants.

At the synthesis stage, it was observed that depending on the product average polyaddition degree and the applied catalyst, the distinctly different molecular weight range of the homologues (MWD) can be obtained. Lower MWD values of polyoxyalkylation products obtained with the DMC catalyst indicated narrower molar mass ranges compared to those synthesized using KOH. Furthermore, it was determined that the polydispersity of the tested products decreases when increasing the average molecular weights.

In terms of surface activity, it was concluded that all synthesized derivatives of 2-ethylhexanol exhibited similar adsorption capacities at the air/water interface and a comparable propensity for micellization after saturation of the interface. On the other hand, the EHP1E9 derivative is characterized by a higher CMC value compared to the considered commercial surfactants and at a concentration of CMC, this non-ionic surfactant lowers the surface tension of water more effectively than the other three commercial formulations. Moreover, the commercial preparations reveal a lower tendency toward adsorption at the air/aqueous solution interface (which is confirmed by much lower ΔG_ads_ values of synthesized derivatives), but simultaneously, the commercial formulations form a much more densely populated adsorption monolayer at the saturated interface. The study also revealed that the superior absorption of the sterically specific amphiphilic structure of synthesized surfactants at the glass/water interphase significantly reduces the concentration (availability) of these surfactants in the aqueous solution.

A new algorithm useful to determine the diffusion coefficients of surfactants based on the experimental data of the dynamic adsorption process was presented. This algorithm revealed that the diffusivity of new surfactants derived from 2-ethylhexanol estimated on the basis of the developed algorithm is about two to three rows of magnitude higher as compared to the commercial preparations. It has been experimentally demonstrated that all synthesized derivatives of 2-ethylohexanol represent the group of very good wetting agents, especially with regard to the polymer surface.

In summary, the innovative surfactants described in this article, derived from 2-EH alcohol and synthesized using dimetalcyanide catalysts, demonstrate considerable potential to compete with conventional surfactant preparations such as Dodecylbenze-nesulfonic Acid (ABS) or Sodium Alcohol Ether Sulphate (AES) acid salts.

## Figures and Tables

**Table 1:** The range of average molecular weights and calculated MWD dispersity parameter of counterparts with the same average polyaddition degree, depending on the catalyst used.

Analyzed Surfactant, Average Molar Ratio/ Catalyst Type	EH-P_1_E_3_-/KOH	EH-P_1_E_3_-/DMC	EH-P_1_E_9_-/KOH	EH-P_1_E_9_-/DMC
Mw—weight balance	320 Da	320 Da	584 Da	584 Da
Analyzed surfactant, synthesis mass balance	EH = 40.6% P1 = 18.1% E3 = 41.3%		EH = 22.3% P1 = 9.9% E9 = 67.8%	
Mw at 1/2 peak height—start (Retention time)	822 Da (13.87 min)	487 Da (15.06 min)	956 Da (13.50 min)	740 Da (14.12 min)
Mw at 1/2 peak height—end (Retention time)	180 Da (17.05 min)	249 Da (16.44 min)	541 Da (14.83 min)	532 Da (14.87 min)
Polydispersity, MWD	1.34	1.12	1.16	1.05

**Table 2:** The values of parameters characterizing the adsorption properties of 2-EH alcohol-derived nonionic and anionic surfactants and commercial compounds.

Surfactant	CMC	pC_20_	Π_CMC_	C	k_2_	τ_2_
	(mmol/L)	(—)	(mN/m)	(~1CMC)	(s^−1^)	(ms)
EHP_1_E_9_	9.40	3.96	41.24	11.08	0.58	1736.20
SSEHP_1_E_9_	4.81	3.55	41.11	5.25	0.84	1191.19
SSEHP1E3	5.62	3.55	41.94	8.75	0.50	2000.19
SSEHP_3_E_9_	3.40	3.85	43.16	5.25	0.91	1095.96
SSEHP_3_E_3_	4.39	3.85	42.92	5.25	1.66	602.19
Triton X100	0.34	4.70	42.40	0.40	1.61	622.64
SDS	8.09	2.60	35.17	8.50	2.99	334.33
ABSNa	2.00	3.30	37.64	3.00	1.24	803.90
SLES25	0.75	4.00	39.97	0.80	2.55	392.70

**Table 3:** The values of Szyszkowski equation coefficients (A_sz_ i B_sz_) and adsorption parameters (ΔG_ads_, Γ^∞^, A_min_) for commercial compounds and synthesized derivatives.

Surfactant	A_sz_∙10^6^ (L/mol)	B_sz_ (-)	−ΔG_ads_ (kJ/mol)	Γ^∞^ × 10^6^ (mol/m^2^)	A_min_ (nm^2^)
ABSNa	2.43 × 10^−4^	0.24	20.34	7.17	0.23
SLES25	2.71 × 10^−5^	0.17	25.84	5.12	0.32
EHP_1_E_9_	7.53 × 10^−7^	0.06	34.46	1.88	0.88
SSEHP_1_E_9_	2.94 × 10^−6^	0.07	31.13	2.18	0.76
SSEHP_3_E_3_	5.41 × 10^−6^	0.09	29.64	2.78	0.59
SSEHP_1_E_3_	1.14 × 10^−5^	0.09	27.83	2.72	0.61

**Table 4:** Descriptions of the tested innovative EH-based surfactants.

Surfactant Symbol	Description
	Nonionic and anionic, a-propoxy, n-ethoxy 2-EH—surfactant:
EHP_1_E_9_	Mono-propoxylated 2-ethylhexanol polyethoxylate of average addition degree *n* = 9, DMC catalyst
	Anionic, sulfosuccinic surfactants:
SSEHP_1_E_9_	Mono-propoxylated 2-ethylhexanol polyethoxylate of average addition degree *n* = 9, DMC catalyst, sulfosuccinate sodium salt
SSEHP_1_E_3_	Mono-propoxylated 2-ethylhexanol polyethoxylate of average addition degree *n* = 3, DMC catalyst, sulfosuccinate sodium salt
SSEHP_3_E_9_	Three-propoxylated 2-ethylhexanol polyethoxylate of average addition degree *n* = 9, DMC catalyst, sulfosuccinete sodium salt
SSEHP_3_E_3_	Three-propoxylated 2-ethylhexanol polyethoxylate of average addition degree *n* = 3, DMC catalyst, sulfosuccinete sodium salt

## Data Availability

The data presented in this study are available in article.
